# Prospects of organ donation among general population in KSA: Promising perceptions but limited commitment

**DOI:** 10.1016/j.jtumed.2026.07.005

**Published:** 2026-08-13

**Authors:** AM. M. Badahdah, Mohammed O. Nassif

**Affiliations:** a Department of Family and Community Medicine, Faculty of Medicine in Rabigh, King Abdulaziz University, Jeddah, Saudi Arabia; b Department of Surgery, Faculty of Medicine, King Abdulaziz University, Jeddah, Saudi Arabia

**Keywords:** الموقف من الصحة, التبرع بالأعضاء, المملكة العربية السعودية, الرغبة في التبرع بالأعضاء, Attitude to health, KSA, Organ donation perception, Willingness to donate organs

## Abstract

**Background:**

Despite high public support, organ donation after death remains suboptimally practiced in Saudi Arabia. This disparity highlights a critical willingness-to-commitment gap in the population. This study aimed to identify a historical baseline of awareness and acceptance of organ donation after death, and explore its principal determinants and barriers.

**Methods:**

Retrospective analysis was conducted using a data set derived from an anonymous cross-sectional survey among adult residents of Saudi Arabia in 2017. The questionnaire assessed awareness of donation campaigns, attitudes toward organ donation after death, and the possession of donor card. Associations between demographic variables and key outcomes were analyzed using chi-squared tests and odds ratios (ORs).

**Results:**

Among 2609 participants (mean age 30.9 ± 11.1 years; 56% female; 94.5% Saudi), 82.1% supported organ donation after death but only 5.6% possessed donor cards. Males expressed lower support (OR = 0.76, *p* < 0.05). Higher educational qualification was associated with increased awareness of donation events (OR = 1.38, *p* < 0.01), knowledge of donor cards (OR = 1.41, *p* < 0.01), and card possession (OR = 2, *p* < 0.01). Being employed significantly impacted awareness and card possession ( *p* < 0.01). Religious uncertainty (26%), emotional fears (20%), and concerns about body manipulation (17%) were the most cited barriers. Males more often cited religious reasons (33%), whereas women more frequently reported personal concerns (23%).

**Conclusion:**

Public support for organ donation is strong in Saudi Arabia but formal registration remains low. To bridge this intention-to-action gap, public health efforts could benefit from targeted outreach directed at key demographic groups characterized by high acceptance, as well as incorporating strategies to reach underserved populations with lower awareness to ensure a more comprehensive public health approach.

## الملخص

**أهداف البحث:**

على الرغم من الدعم الكبير في المجتمع، لا يزال التبرع بالأعضاء بعد الوفاة يُمارس على نحو غير مثالي في المملكة العربية السعودية. يُسلط هذا التفاوت الضوء على فجوة كبيرة في رغبة السكان في الالتزام بهذا الإجراء. هدفت هذه الدراسة إلى رسم خط أساس تاريخي للوعي والقبول بالتبرع بالأعضاء بعد الوفاة، واستكشاف محدداته الرئيسية ومعوقاته.

**طرق البحث:**

تحليل استرجاعي لبيانات مستقاة من مسح مقطعي مجهول الهوية أُجري بين البالغين المقيمين في المملكة العربية السعودية عام ٢٠١٧. قيّم الاستبيان الوعي بحملات التبرع، والاتجاهات نحو التبرع بالأعضاء بعد الوفاة، وحيازة بطاقة التبرع. تم تحليل العلاقات بين المتغيرات الديموغرافية والنتائج الرئيسية باستخدام اختبارات مربع كاي ونسب الأرجحية.

**النتائج:**

بلغ عدد المشاركين ٢٦٠٩ مشاركًا (متوسط العمر ٣٠.٩ ± ١١.١ سنة؛ ٥٦٪ إناث؛ ٩٤.٥٪ سعوديين). على الرغم من أن ٨٢.١٪ منهم أيدوا التبرع بالأعضاء بعد الوفاة، إلا أن ٥.٦٪ فقط منهم كانوا يمتلكون بطاقات التبرع. أبدى الذكور دعمًا أقل. ارتبط ارتفاع مستوى التأهيل بزيادة الوعي بفعاليات التبرع بالأعضاء، ومعرفة بطاقات التبرع، وحيازة البطاقة. كما كان للعمل تأثير كبير على الوعي وحيازة البطاقة. في المقابل، كانت الشكوك الدينية (26%)، والمخاوف العاطفية (20%)، والقلق بشأن التعامل مع الجسد (17%) من أكثر العوائق شيوعًا. ذكر الذكور أسبابًا دينية أكثر (33%)، بينما ذكرت النساء مخاوف شخصية أكثر (23%).

**الاستنتاجات:**

يوجد دعم قوي للتبرع بالأعضاء في المملكة العربية السعودية، ومع ذلك لا يزال التسجيل الرسمي منخفضًا. لسد هذه الفجوة بين النية والفعل، يمكن لجهود الصحة العامة أن تستفيد من حملات توعية موجهة إلى الفئات الديموغرافية الرئيسية التي تُظهر قبولًا واسعًا، مع دمج استراتيجيات للوصول إلى الفئات السكانية المحرومة ذات الوعي المنخفض لضمان اتباع نهج شامل في مجال الصحة العامة.

## Introduction

Since the first successful organ transplant in 1954, thousands of lives have been saved.^1^^,^^2^ However, the global demand continues to vastly outpace supply, where 157,553 solid organ transplants were performed globally in 2022 alone, but this only met less than 10% of the estimated need.^3^ The severity of this deficit is further underscored by recent data from the United States, where in July 2025, more than 10 individuals died each day while awaiting a suitable organ, and approximately 105,000 candidates remained on the national transplant waiting list.^4^ This gap arises not only from economic and medical infrastructural limitations, but also from low donor availability.^5^^,^^6^ Barriers such as limited awareness, ethical uncertainty, religious misconceptions, and fear of medical procedures have hindered public participation in organ donation.^7^

In KSA, organ transplantation began with the first living donation in 1979 and the formalization of organ donation after death (ODAD) in 1985. The Saudi Center for Organ Transplantation (SCOT) was subsequently inaugurated in 1993 and a period of unprecedented transformation has recently started.^8^^,^^9^ Driven by the ambitious healthcare reforms of Saudi Vision 2030, the SCOT coordinating efforts have achieved progress in living donor transplantation, positioning the Kingdom third globally with a rate of 45.49 per million population.^9^, ^10^, ^11^ By 2023, over 200 individuals had received the prestigious King Abdulaziz Medal for exceptional contributions to organ donation.^12^ However, understanding the true magnitude of these recent national advances requires a clear view of the pre-reform landscape.

Despite these successes and institutional efforts, deceased donor registration within KSA's opt-in system remains critically low, averaging just 3.31 per million population.^10^ Operationally, the national framework requires next-of-kin approval before any procurement procedure can take place. Historical utilization of reported deceased donor cases is inefficient, and a regional report indicated that only 6% of reported cases were utilized, with family refusal rates between 1986 and 2022 estimated at a staggering 67%.^13^ This persistent challenge highlights a significant disparity between the actual act and public acceptance of donation.

Public engagement with organ donation in KSA is on the rise. Historical and recent national and urban surveys have generally reported high acceptance rates (74% to over 87%),^14^, ^15^, ^16^, ^17^, ^18^, ^19^, ^20^, ^21^ and a strong willingness to donate (up to 77.7%).^14^, ^15^, ^16^^,^^22^^,^^23^ Studies among healthcare professionals indicate similarly high acceptance (up to 85%) and willingness rates (up to 92%).^24^, ^25^, ^26^ By contrast, peripheral regions exhibit lower engagement, with acceptance sometimes as low as 42%^27^, ^28^, ^29^ and willingness dropping to 30.3%.^30^^,^^31^ Despite these promising figures, formal registration historically remains critically low, where donor card possession is typically below 6%, with rates as low as 1.4% reported in regions such as Qassim. Even among medical professionals, the registration rate of 33.6% reflects a persistent “willingness-to-commitment” gap that was embedded in the community prior to modern interventions, and necessitates further investigation.^21^^,^^25^, ^26^, ^27^^,^^32^^,^^33^

Frameworks such as the Health Belief Model (HBM), Theory of Reasoned Action (TRA), and Theory of Planned Behavior (TPB) are widely used to categorize the factors that shape organ donation behaviors. In particular, the HBM evaluates decisions by weighing perceived benefits against barriers, such as religious misconceptions or procedural fears. In addition, the TRA and TPB emphasize the influences of subjective norms (e.g., family acceptance) and perceived behavioral control (e.g., accessibility of registration).

KSA is continuing to modernize its healthcare infrastructure under the Vision 2030 framework, and evaluating the progress of these reforms requires a historical benchmark. Hence, this study applied a national data set from 2017 to establish a pre-reform baseline and identify the socioeconomic factors and psychological barriers that predict or prevent ODAD awareness, support, and card possession among individuals.

## Materials and Methods

This study involved retrospectively analyzing a foundational data set originally collected via an anonymous cross-sectional survey distributed among residents of KSA between March and May 2017. To establish a pre-vision 2030 snapshot, this study aimed to: (a) assess public awareness and exposure to organ donation campaigns, (b) measure support and positive perceptions toward donation after death, (c) determine the prevalence of organ donor card possession, and (d) examine how these factors vary across demographic groups.

### Data source and variables

The analyzed data set was derived from a self-administered Arabic questionnaire designed to be accessible to an appropriate reading level. Content validity was established through a pilot test involving 15 adults, where their feedback helped to refine the questionnaire's linguistic clarity, logical flow, and cultural appropriateness prior to deployment.

For this retrospective analysis, data were extracted across three primary domains: (a) socio-demographic characteristics (e.g., age, gender, education level, employment status, and region of residence); (b) awareness and commitment, including public awareness of and exposure to organ donation campaigns, the prevalence of organ donor card possession, and baseline support for ODAD; and (c) reasons for non-support, where participants who indicated “No” to the preceding support question (“Do you support organ donation after death?”) were subsequently prompted with “If 'No’, Why?” and provided with options to specify their primary rationale from a predefined list. Responses were securely collected through a web-based form hosted by SurveyMonkey™ (SurveyMonkey Inc., San Mateo, California, USA), and all data were exported as a Microsoft Excel™ file for analysis.

### Participant recruitment and sampling

The original convenience sample was recruited via a web-based form disseminated through major social media platforms and widely used communication applications. Eligible participants were adult residents (citizens and non-citizens) of KSA. The cover page of the questionnaire served as the consent form, informing participants about the study's purpose, confidentiality, and voluntary nature. Submission of the completed survey was considered implied consent. No personal identifying data were collected. This study was reviewed and approved by the Institutional Review Board of King Abdulaziz University, Jeddah, Saudi Arabia.

### Statistical analysis

Descriptive statistics were used to characterize the study population and report key outcomes (such as awareness, support, and card possession) as proportions based on the number of valid (non-missing) responses for each respective question. To identify associations between various demographic predictors and these key outcomes, odds ratios (ORs) were calculated with 95% confidence intervals, and multivariable logistic regression was performed. Pearson's chi-squared test was utilized to compare categorical demographic variables and key outcomes. Responses from participants who stated “unsure” or “I do not know” to key questions were excluded from the analysis for OR calculations and logistic regression. The percentage distribution of reported reasons for non-support was analyzed and stratified by gender. A *p*-value ≤0.05 was considered to indicate a statistically significant difference. All statistical analyses were performed using the Statistical Package for Social Sciences (SPSS) for Windows v.25.0 (IBM Corp., Armonk, NY, USA).

## Results

The study sample comprised 2609 adults with a mean age of 30.87 ± 11.14 years and a modest female predominance (ratio of 1.34:1). The majority were Saudi nationals (94.5%). Geographically, nearly half of the participants resided in Makkah Province (47.8%), followed by Riyadh (23.6%). Educational levels were high, with only 0.3% reporting no formal education. Most participants held either a diploma or a bachelor's degree (68.7%), while 19.2% had a postgraduate qualification. Most participants (69.2%) were employed, where the remainder comprised students, retirees, housewives, and the unemployed (Table 1).

**Table 1:** Demographic characteristics of participants.

Characteristics	All *n*/*N*^a^ (%)
**Number of participants**	2609
**Age in years**	
Mean (SD)	30.87 (±11.14)
Range (Median)	18–76 (27)
**Gender**	
Female:male	1.34:1
**Nationality**	
KSA	2460/2603 (94.51)
Other	143/2603 (5.49)
**Province of residence**	
Makkah	1244/2604 (47.77)
Riyadh	615/2604 (23.62)
Eastern	315/2604 (12.10)
Al-Madinah	147/2604 (5.65)
Al-Qassim	90/2604 (3.46)
Assir	68/2604 (2.61)
Other	125/2604 (4.80)
**Highest qualification**	
No formal education	7/2591 (0.27)
School certificate^b^	305/2591 (11.77)
Diploma/bachelor's degree	1781/2591 (68.74)
Postgraduate degree	498/2591 (19.22)
**Employed**	
No^c^	800/2598 (30.79)
Yes	1798/2598 (69.21)

SD - standard deviation.

a The total number of respondents with complete information for each individual variable.

b Completion of secondary education.

c Includes students, retired and housewives.

Initial unadjusted analysis indicated consistently high support for ODAD across all demographic groups, with an overall support rate of 82.1%. Males reported lower support compared with females (79.9% vs. 83.9%; OR = 0.75, *p* < 0.05). Furthermore, 84.5% of the sample supported adding donation authorization to official documents. Despite the high overall support, only 5.5% of the total sample possessed a donor card, revealing a prominent willingness-to-commitment gap. No substantial differences in general support were observed by nationality, province of residence, education level, or employment status, where the support rates among these groups ranged from 81% to 83% (Table 2).

**Table 2:** Awareness, acceptance, and support for organ donation.

Characteristics	Heard of organ donation event			Heard about organ donation card			Support organ donation after death			Carrying an organ donation card			Support adding organ donation authorisation to official documents		
	*n*/*N*^a^ (%)	OR (95% CI)	*p*	*n*/*N*^a^ (%)	OR (95% CI)	*p*	*n*/*N*^a^ (%)	OR (95% CI)	*p*	*n*/*N*^a^ (%)	OR (95% CI)	*p*	*n*/*N*^a^ (%)	OR (95% CI)	*p*
**All**															
All participants^¶^	1633/2607 (62.6)			1467/2601 (56.4)			2138/2604 (82.1)			145/2607 (5.6)			2192/2593 (84.5)		
**Sex**															
Female	984/1490 (66.0)	1.0 (ref)		901/1486 (60.6)	1.0 (ref)		1247/1487 (83.9)	1.0 (ref)		105/1490 (7.1)	1.0 (ref)		1279/1481 (86.4)	1.0 (ref)	
Male	646/1114 (58.0)	0.71 (0.60–0.83)	<0.001^b^	565/1112 (50.8)	0.67 (0.57–0.79)	<0.001^b^	890/1114 (79.9)	0.75 (0.62–0.94)	<0.05^b^	40/1114 (3.6)	0.49 (0.34–0.71)	<0.001^b^	911/1109 (82.2)	0.73 (0.59–0.90)	0.003^b^
**Nationality**															
Other	83/143 (58.0)	1.0 (ref)		61/143 (42.7)	1.0 (ref)		119/143 (83.20)	1.0 (ref)		5/143 (3.5)	1.0 (ref)		119/141 (84.4)	1.0 (ref)	
KSA	1546/2458 (62.9)	1.23 (0.87–1.73)	0.24	1405/2452 (57.3)	1.8 (1.28–2.54)	<0.001^b^	2013/2455 (82.0)	0.92 (0.59–1.44)	0.711	140/2458 (5.7)	1.67 (0.67–4.13)	0.265	2068/2446 (84.6)	1.01 (0.63–1.61)	0.962
**Province of residence**															
Other	858/1359 (63.1)	1.0 (ref)		799/1238 (53.7)	1.0 (ref)		1122/1356 (82.7)	1.0 (ref)		80/1360 (5.9)	1.0 (ref)		1130/1347 (83.9)	1.0 (ref)	
Makkah province	773/1243 (62.2)	0.96 (0.82–1.13)	0.62	665/1358 (58.8)	0.81 (0.70–0.95)	<0.01^b^	1012/1243 (81.4)	0.90 (0.75–1.12)	0.378	65/1242 (5.2)	0.88 (0.63–1.24)	0.471	1057/1241 (85.2)	1.10 (0.89–1.37)	0.376
**Postgraduate qualification**															
No	1278/2092 (61.1)	1.0 (ref)		1143/2087 (54.8)	1.0 (ref)		1717/2089 (82.2)	1.0 (ref)		99/2091 (4.7)	1.0 (ref)		1743/2079 (83.8)	1.0 (ref)	
Yes	343/497 (69.0)	1.42 (1.15–1.75)	0.001^b^	315/496 (63.5)	1.44 (1.17–1.76)	<0.001^b^	407/497 (81.9)	0.98 (0.76–1.26)	0.875	46/498 (9.2)	2.05 (1.42–2.95)	<0.001^b^	434/496 (87.5)	1.35 (1.01–1.80)	0.043^b^
**Employed**															
No	471/800 (58.9)	1.0 (ref)		409/798 (51.3)	1.0 (ref)		667/799 (83.5)	1.0 (ref)		31/799 (3.9)	1.0 (ref)		680/795 (85.5)	1.0 (ref)	
Yes	1154/1796 (64.3)	1.26 (1.06–1.49)	<0.01^b^	1052/1792 (58.7)	1.35 (1.14–1.60)	<0.001^b^	1460/1794 (81.4)	0.87 (0.69–1.08)	0.199	111/1797 (6.2)	1.63 (1.09–2.45)	<0.05^b^	1501/1787 (84.0)	0.89 (0.70–1.12)	0.319

OR, odds ratio; 95% CI, 95% confidence interval; *p*, *p-*value; ref, reference value.

a The total number of respondents with complete information for both variable in comparison.

b Statistically significant.

According to the multivariable logistic regression model (Table 3), participants with a postgraduate qualification had higher odds of being aware of donation events (OR 1.38 [1.11–1.72], *p* = 0.003) and organ donation cards (OR 1.41 [1.14–1.74], *p* = 0.001), as well as carrying an organ donation card (OR 2.00 [1.37–2.92], *p* < 0.001) and supporting the inclusion of authorization in official documents (OR 1.40 [1.04–1.89], *p* = 0.026). Similarly, employed participants had higher odds of awareness regarding donation events (OR 1.37 [1.14–1.65], *p* < 0.001) and cards (OR 1.52 [1.27–1.83], *p* < 0.001), and were more likely to possess a donation card (OR 1.80 [1.17–2.76], *p* = 0.008). Compared with females, male participants had lower odds of being aware of donation events (OR 0.64 [0.54–0.76], *p* < 0.001) and donation cards (OR 0.57 [0.48–0.67], *p* < 0.001). Males also had lower odds of supporting ODAD (OR 0.76 [0.61–0.94], *p* = 0.012), carrying a donation card (OR 0.41 [0.28–0.61], *p* < 0.001), and supporting the inclusion of authorization in official documents (OR 0.76 [0.60–0.95], *p* = 0.016). Regarding nationality and region of residence, Saudi citizens had higher odds of awareness of organ donation cards (OR 1.76 [1.24–2.50], *p* = 0.002), whereas residing in Makkah Province was associated with lower odds of awareness of donation cards (OR 0.72 [0.62–0.85], *p* < 0.001).

**Table 3:** Logistic analysis regression for sociodemographic predictors of attitudes toward study outcomes.

Characteristic	Heard of organ donation event				Heard about organ donation card				Support organ donation after death				Carrying an organ donation card				Support adding organ donation authorisation to official documents			
		95% CI				95% CI				95% CI				95% CI				95% CI		
	OR	Lower	Upper	*p*-value	OR	Lower	Upper	*p*-value	OR	Lower	Upper	*p*-value	OR	Lower	Upper	*p*-value	OR	Lower	Upper	*p*-value
Male gender	0.64	0.54	0.76	<0.001^a^	0.57	0.48	0.67	<0.001^a^	0.76	0.61	0.94	0.012^a^	0.41	0.28	0.61	<0.001^a^	0.76	0.60	0.95	0.016^a^
Saudi citizenship	1.15	0.81	1.63	0.448	1.76	1.24	2.50	0.002^a^	0.91	0.57	1.44	0.673	1.48	0.59	3.70	0.404	1.07	0.67	1.72	0.776
Residence of Makkah province	0.87	0.74	1.03	0.104	0.72	0.62	0.85	<0.001^a^	0.88	0.71	1.08	0.209	0.71	0.50	1.01	0.055	1.05	0.84	1.31	0.682
Postgraduate qualification	1.38	1.11	1.72	0.003^a^	1.41	1.14	1.74	0.001^a^	1.03	0.79	1.34	0.831	2.00	1.37	2.92	<0.001^a^	1.40	1.04	1.89	0.026^a^
Employed	1.37	1.14	1.65	<0.001^a^	1.52	1.27	1.83	<0.001^a^	0.93	0.73	1.18	0.547	1.80	1.17	2.76	0.008^a^	0.90	0.70	1.16	0.432

95% CI; 95% confidence interval; OR, odds ratio.

a Statistically significant.

**Figure 1:**
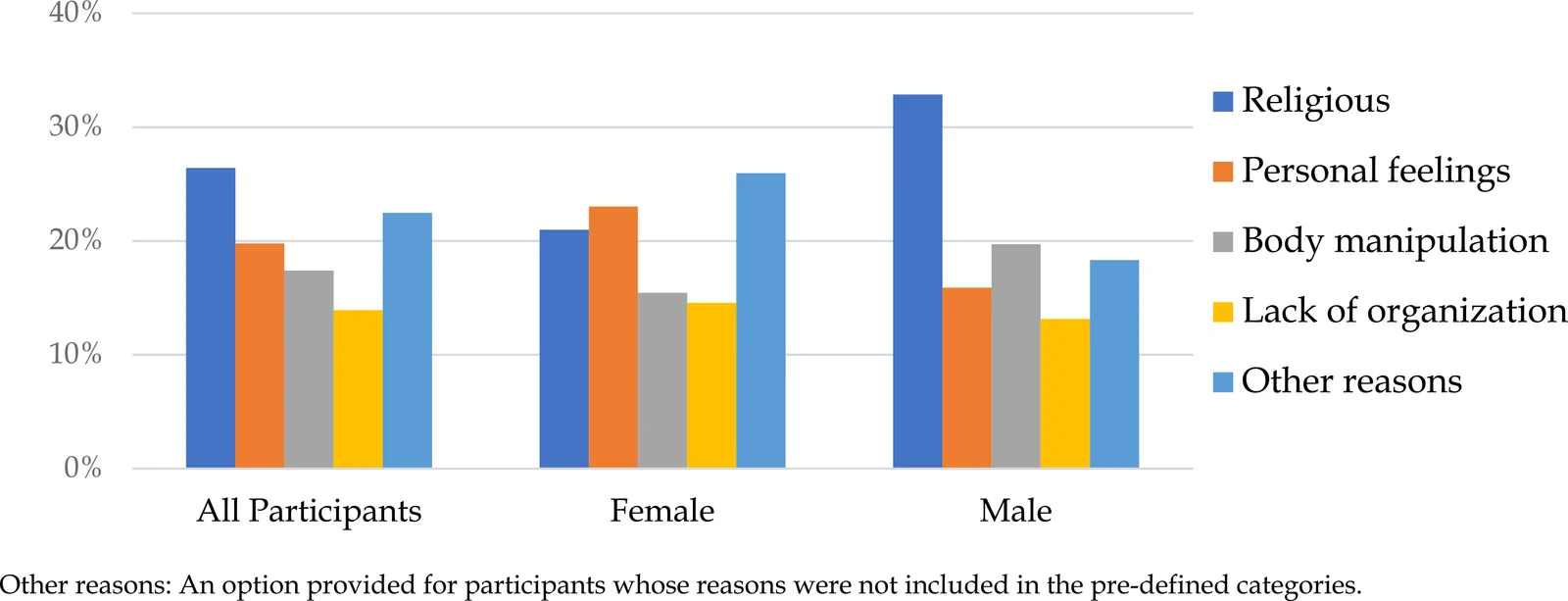
Main reasons preventing non-supporting participants from supporting organ donation after death (n = 464).

Among those who did not support ODAD, a range of concerns emerged, highlighting specific emotional, religious, and procedural factors that influenced their stance. As illustrated in Figure 1, religious concerns were the most frequently cited concerns (26%), followed by unspecified “other” reasons (22%) and personal feelings (20%). Gender-stratified analysis revealed that religious concerns were more common among male participants (33%), whereas females more frequently cited personal feelings (23%) and unspecified “other” reasons (26%). Religious concerns and personal feelings were the main causes reported for not supporting ODAD among all participants, with a slightly higher prevalence in males (20%) compared with females (15%). Lack of organizational clarity was the least cited barrier (14%) and there was minimal variation by gender.

## Discussion

This study demonstrated high public awareness and support (82.1%) for organ donation in KSA. Key demographics (women, postgraduates, and employees) had positive attitudes, but religious concerns and personal feelings were reported as barriers by the non-supporting minority. Crucially, despite this acceptance rate, actual commitment remains critically low, with only 5.6% carrying a donor card. This significant discrepancy between intent and behavior potentially indicates a classic “intention–behavior gap,” where high motivation fails to manifest as formal commitment due to limited “perceived behavioral control” and procedural friction.

### Awareness, acceptance, and predictors

The high rate of support suggests the presence of strong “subjective norms” consistent with recent nationwide studies (87.4% in 2019 and 87% in 2023),^14^^,^^17^ suggesting that public health campaigns have established subjective norms that endorse organ donation as a pro-social behavior.

Female participants consistently exhibited higher awareness across all outcomes. This engagement aligns with current findings in Bisha and Al-Qassim,^27^^,^^28^ and may stem from women's traditional life-giving and caregiving roles, which could explain this altruistic health behavior.^34^^,^^35^ However, the translation of this awareness into practice is still mediated by the pivotal role of male consent in family decision making, necessitating tailored, culturally endorsed messaging to address their specific concerns.

Educational attainment, particularly at the postgraduate level, and active employment were strongly associated with ODAD awareness and support, consistent with established Saudi trends.^17^^,^^27^^,^^30^ Employment often reflects these educational advantages, so this socioeconomic group represents the demographic that is most likely to support donation and possess donor cards. The supportive majority reported high perceived benefits, whereas the hesitant minority had a distinct cognitive profile. A comprehensive analysis of the Saudi landscape necessitates examining the contrasting hesitant minority, whose opposition is primarily rooted in socio-cultural, emotional, and theological concerns.

### Barriers to positive perception

Among the non-supportive minority, religious uncertainty, emotional fears, and concerns regarding bodily integrity were the primary barriers to ODAD acceptance. These concerns had distinct gendered patterns, necessitating segmented messaging. Within the HBM framework, these factors function as formidable perceived barriers that outweigh the perceived benefits of donation, signaling a shift toward belief-restructuring interventions rather than traditional awareness campaigns. The present analysis quantified the prevalence of these obstacles but the data set lacked the granularity required to interpret the underlying drivers. Thus, these findings may serve as a baseline for future qualitative research to investigate these complexities and inform more targeted interventions.

### Commitment and card possession

Despite a solid foundation of support, the observed registration rate of 5.6% aligns with regional data from 2016 in Jeddah (4.6%) and Almadinah Almunawwarah (2.7%).^21^^,^^33^ Some recent national reports cite higher registration figures ranging from 17.8% to 21% in the general population^17^^,^^36^ and reaching 33.6% among healthcare professionals,^26^ but these figures still contradict another recent finding of a registration rate of 5.4% in 2023, which indicates a profound intention–behavior gap. In our cohort, almost 93% of those supporting ODAD failed to declare card possession, echoing previous findings where 84–98% of those supporting or willing to donate participants failed to formalize their commitment.^21^^,^^27^^,^^32^^,^^33^ The present study did not qualitatively evaluate specific motives for non-registration, but this disparity could be explained by systemic and logistical obstacles. For instance, data from Al-Kharj indicate that 97% of participants were unaware of the registration process despite their support.^37^ Other reports list ambiguity regarding registration procedures, concerns over organ misuse, or misconceptions regarding brain death.^19^^,^^26^ These obstacles diminish perceived behavioral control, making the act of registration feel inaccessible. Ultimately, the conversion of supportive intent into official registration represents a challenge for the Saudi transplantation community.

Insights into demographic variations in ODAD card possession provide a clearer understanding of the drivers of potential conversion. Female gender, postgraduate education, and active employment were associated with an increase of at least 1.8-fold in the likelihood of possessing an ODAD card. This suggests that educational attainment is closely linked to health literacy and the capacity for institutional navigation, which are both key drivers of translating attitudes into action. Building on these demographic associations, professional environments emerge as high-yield avenues for advancing organ donor registration.

Workplaces are ideal settings for general health promotion^38^ and vital for organ donor initiatives. High-intensity campaigns and integrating media with interpersonal communication have consistently led to substantial increases in employee registration rates.^39^^,^^40^ In particular, the use of “message immediacy”^41^ and perceived workplace support^42^ can amplify campaign impacts. International successes often rely on “opt-out” systems, but a modified approach for the Saudi context could involve an “active-choice” model. Studies suggest that by optimizing the choice architecture within these professional environments, the supportive population can be prompted to complete registration at the point of employment. Thus, maintaining a clear, perpetual option to opt out acts as a behavioral nudge that bypasses procedural and cognitive friction to bridge the intent–action gap.

Our findings and those obtained in other studies highlight the substantial progress made in improving public awareness and positive attitudes toward organ donation in KSA. The primary challenge is effectively converting widespread willingness into documented commitment, which necessitates a clear strategic shift toward institutional facilitation to motivate the willing majority. To realize this strategic shift, we recommend: (a) deploying mobile and workplace registration units with digital reminders, (b) shifting resources from broad awareness to process facilitation and trust-building initiatives, and (c) monitoring conversion metrics and adapting outreach by gender and education level.

### Limitations

This study provides valuable insights, but our findings must be interpreted in context. The cross-sectional data were used to establish correlations rather than causality; therefore, contemporary replication is warranted. Our sample was geographically diverse, but disproportionately represented by participants from two regions (over 75%), limiting broader regional insights. The web-based convenience sample also skewed toward higher education levels, potentially overestimating awareness and favorable attitudes compared with the general population. Utilizing multi-channel recruitment strategies and adopting validated study instruments are both necessary to achieve broader representation and establish reliability metrics. To overcome measurement constraints, validating self-reported registration and incorporating qualitative insights could further strengthen our findings, and provide a deeper understanding of the emotional and cultural factors that influence organ donation.

## Conclusion

High levels of public awareness and acceptance of organ donation were observed across diverse demographic groups in KSA, revealing a distinct gap between favorable attitudes and official registration. To bridge this intention-to-action gap, literature supports simplifying the registration process and implementing targeted outreach in educational and workplace settings. Public health initiatives should adopt these strategies to facilitate formal registration. Moreover, qualitative research is also necessary to identify the precise barriers that hinder registration, enabling the translation of positive public attitudes into tangible action.

## Ethical approval

This study was reviewed and approved by the Institutional Review Board of King AbdulAziz University, Jeddah, KSA.

## Authors contributions

The manuscript has been read and approved by all the authors, the requirements for authorship have been met by all authors, and each author believes that the manuscript represents honest work. All authors have made substantial contributions to this manuscript: conceptualization, AB and ON; data curation, ON; formal analysis, AB; supervision, ON; validation, AB and ON; writing—original draft, AB; writing—review and editing, AB and ON. All authors have critically reviewed and approved the final draft and are responsible for the content and similarity index of the manuscript.

## Source of funding

This project was funded by the Deanship of Scientific Research (DSR) at King Abdulaziz University, Jeddah, under grant no. G-354-828-38. The authors thank the DSR for technical and financial support.

## Conflict of interest

The authors have no conflict of interest to declare.
